# Molecular analysis of exon 7 of the fibroblast growth factor receptor 2 (*FGFR2*) gene in an Indonesian patient with Apert syndrome: a case report

**DOI:** 10.1186/s13256-019-2173-x

**Published:** 2019-08-07

**Authors:** Gara Samara Brajadenta, Ariestya Indah Permata Sari, Donny Nauphar, Tiar Masykuroh Pratamawati, Vincent Thoreau

**Affiliations:** 1 Department of Medical Biology, Division of Human Genetics, Faculty of Medicine, Swadaya Gunung Jati University, Jalan Terusan Pemuda No.1A, Cirebon, West Java 45132 Indonesia; 2 EA3808 Neurovascular Unit and Cognitive Impairments, University of Poitiers Pole Biologie - Sante (B.36), 1, rue Georges Bonnet, 86073 Poitiers Cedex, France

**Keywords:** Apert syndrome, *FGFR2* mutation, Indonesian patient

## Abstract

**Background:**

Apert syndrome, Online Mendelian Inheritance in Man number 101200, is a rare genetic condition, with autosomal dominant inheritance, characterized by craniosynostosis, midfacial malformation, and severe symmetrical syndactyly. Apert syndrome is associated with other systemic malformations, including intellectual disability. At least seven mutations in fibroblast growth factor receptor 2 (*FGFR2*) gene have been found to cause Apert syndrome. Most cases of Apert syndrome are caused by one of the two most frequent mutations located in exon 7 (Ser252Trp or Pro253Arg).

**Case presentation:**

A 27-year-old Javanese man presented borderline intellectual functioning and striking dysmorphisms. A clinical diagnosis of Apert syndrome was previously made based on these clinical features. Furthermore, POSSUM software was used before molecular analysis and the result showed suspected Apert syndrome with a cut-off point of 14. Molecular genetic analysis of *FGFR2*, targeting exon 7, was performed by direct sequencing. In this patient, a missense mutation c.755C>G was detected, changing a serine into a tryptophan (p.Ser252Trp).

**Conclusion:**

We report the case of an Indonesian man with Apert syndrome with a c.755C>G (p.Ser252Trp) mutation in the *FGFR2* gene. Our patient showed similar dysmorphism to previously reported cases, although cleft palate as a typical feature for p.Ser252Trp mutation was not present. In spite of the accessibility of molecular genetic testing in a few parts of the world, the acknowledgement of clinically well-defined syndromes will remain exceptionally imperative in developing countries with a lack of diagnostic facilities.

## Background

Apert syndrome (AS), Online Mendelian Inheritance in Man (OMIM) 101200, is a rare genetic condition that was first described by Wheaton in 1894 and then by French pediatrician, Dr Eugene Charles Apert, who reported a summary on nine cases in 1906 [1]. It presents with craniosynostosis (premature fusion of the cranial sutures) and acrocephaly, including brachycephaly, midfacial hypoplasia, and syndactyly of hands and feet [1]. Patients exhibit varying degrees of cerebral, cardiac, tracheal, and genitourinary malformations [2]. Additional skeletal manifestations, abnormalities of the skin, and intellectual disability occur at lower frequency [2–6]. An advanced paternal age effect (PAE) has been consistently noted as a factor although most cases of AS are sporadic. However, autosomal dominant inheritance has been widely documented in families [7–9]. The birth prevalence of AS has been reported to be between 1:50,000 and 1:80,000; the highest prevalence has been reported in Asian populations [10].

The fibroblast growth factor receptor 2 (*FGFR2*) gene, OMIM 176943, located on chromosome 10q26, is involved in AS. In 1995, Wilkie *et al.* identified the presence of genetic mutations in exon 7 of the *FGFR2* gene in 40 unrelated patients with AS: c.755C>G (p.Ser252Trp) or c.758C>G (p.Pro253Arg) [11]. These mutations are the most frequent mutations, detected in approximately 85% and 15% of patients diagnosed as having AS, respectively [12, 13].

In this case report, we describe radiological and clinical features present in an Indonesian patient diagnosed as having AS. Moreover, we detected by molecular analysis the presence of the c.755C>G (p.Ser252Trp) mutation in the *FGFR2* gene of this patient.

## Case presentation

Our patient was a 27-year-old Javanese man with borderline intellectual functioning and striking dysmorphisms. Both his parents were Javanese, normal, non-consanguineous, and in their sixth decade of life. He was the third child born after a normal third pregnancy and he had two sisters who were normal. His mother had a cesarean delivery with no history of trauma, infection, or drug use during the term. No family history of similar complaints or any other congenital abnormality was reported. Our patient was born at term after an uneventful pregnancy.

**Fig. 1:**
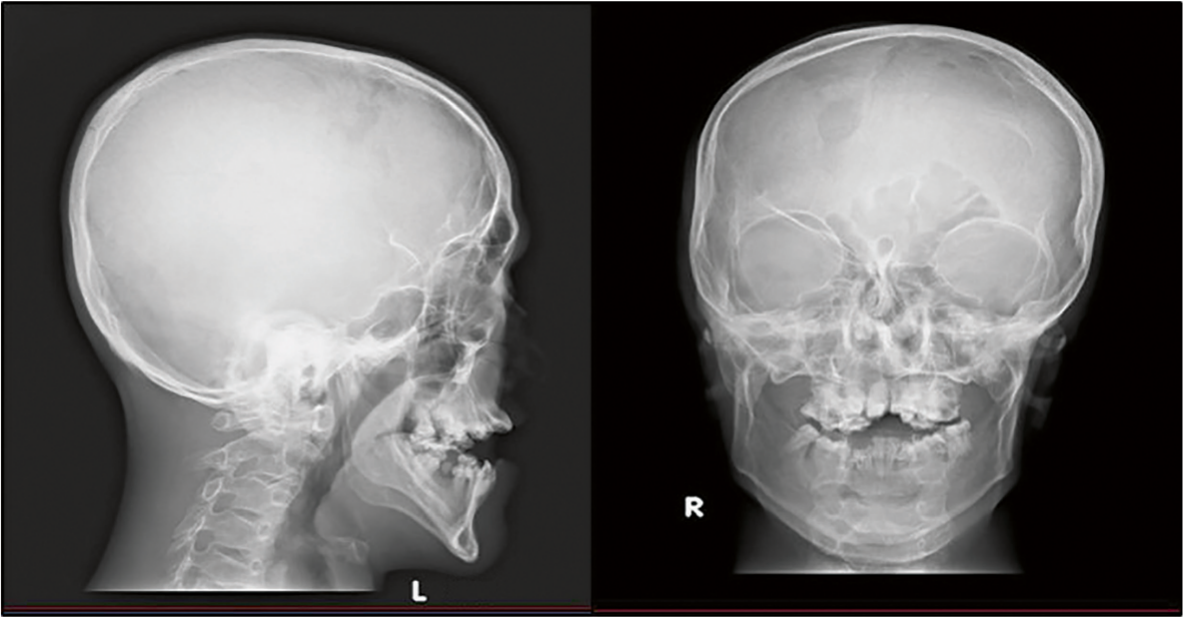
Craniofacial radiography of the patient. Radiographs showing facial dysmorphisms which include acrocephaly, asymmetrical flat facies, nasal deformity, and prominent jaw

**Fig. 2:**
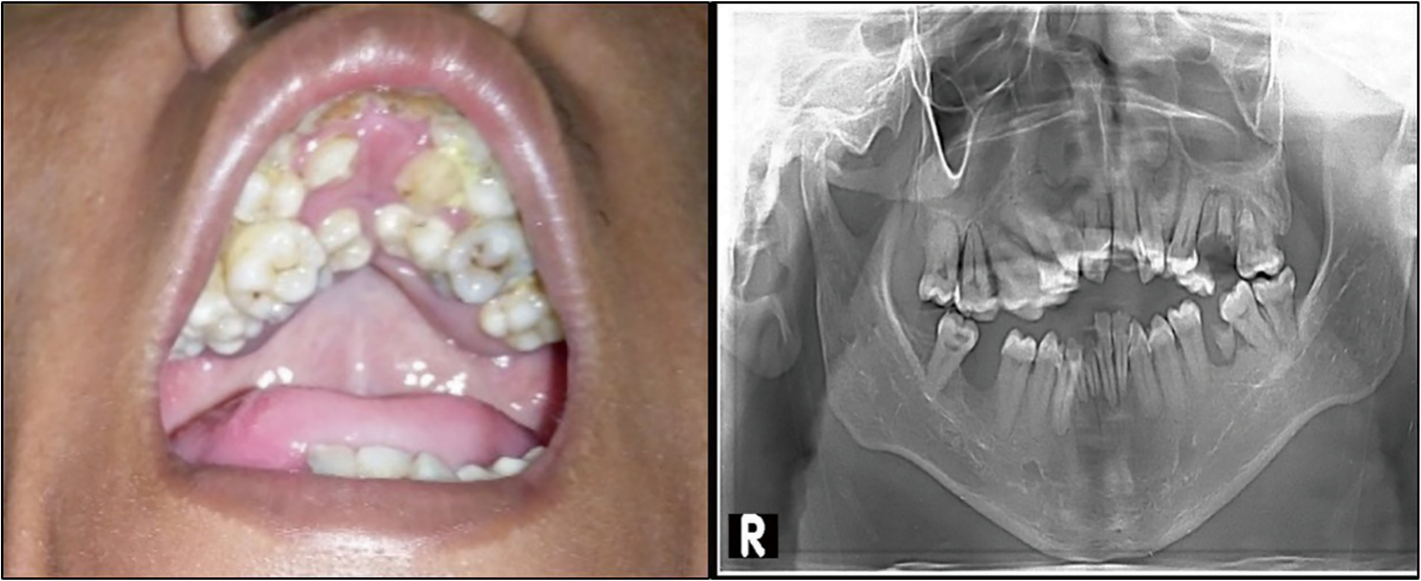
Intraoral appearance of the patient. Photograph showing V-shaped maxillary arch, crowding of teeth, prominent jaw, and high arch palate. Panoramic X-ray showing hypoplastic and retruded maxilla

**Fig. 3:**
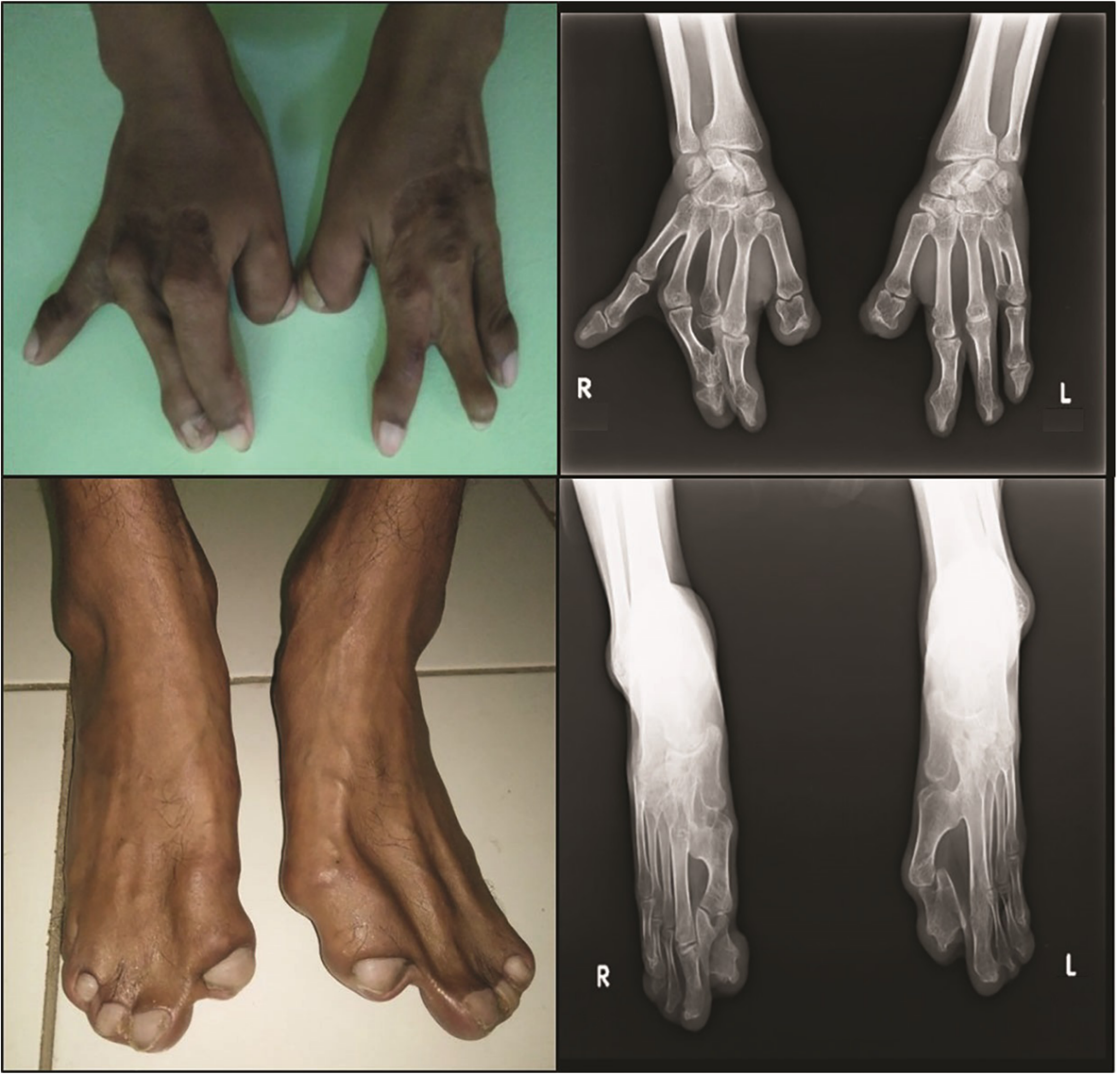
Radiological features of the hands and feet of the patient. Photographs and radiographs showing lack of digit type 2 (fused), symmetrical syndactyly of four digits in both hands (*upper panels*), and type 2 symmetrical syndactyly of five toes in both feet (*lower panels*)

He is a slow learner and attends a school for children with special needs in Cirebon, West Java, Indonesia. There he began to socialize, play with other classmates, and he likes to draw and enjoys music. The dysmorphisms found are very characteristic. On physical examination, his weight was 36 kg, height 158 cm, and occipital frontal circumference 54 cm. It was observed that he displayed hypertelorism, down-slanting palpebral fissure, strabismus, ocular proptosis, depressed nasal bridge, short philtrum, and low-set ears. In addition, acrocephaly, asymmetrical flat facies, nasal deformity, and prominent jaw were present (Fig. 1). His oral deformities showed maxilla hypoplasia with high arch palate. His V-shaped maxillary arch was filled with double rows of teeth. In addition, there was a dental fusion between maxillary premolar and first molar. Panoramic radiographs were performed for confirmation (Fig. 2). Other abnormalities found were mild scoliosis and mild pectus excavatum. Symmetrical cutaneous bilateral syndactyly involving his four fingers, his palms were spoon-shaped with an inwardly placed thumb, was present (type 2). Both feet showed type 2 symmetrical cutaneous syndactyly of the first to fifth toes. Radiographs of both hands and feet confirmed soft tissue syndactyly (Fig. 3). He had corrective surgery twice on both hands to correct for joint contractures. There was no postoperative complication. Six months after the second surgery, he could start using his fingers. A clinical diagnosis of AS was previously made based on these clinical features, as earlier mentioned in our study describing clinical manifestations of this patient [14]. Furthermore, Pictures of Standard Syndromes and Undiagnosed Malformations (POSSUM) software (https://www.possum.net.au/) was used before molecular analysis and the result showed suspected AS with a cut-off point of 14. Ethical clearance for genetic testing was obtained according to the research ethic committee of Faculty of Medicine, Swadaya Gunung Jati University, Indonesia.

**Fig. 4:**
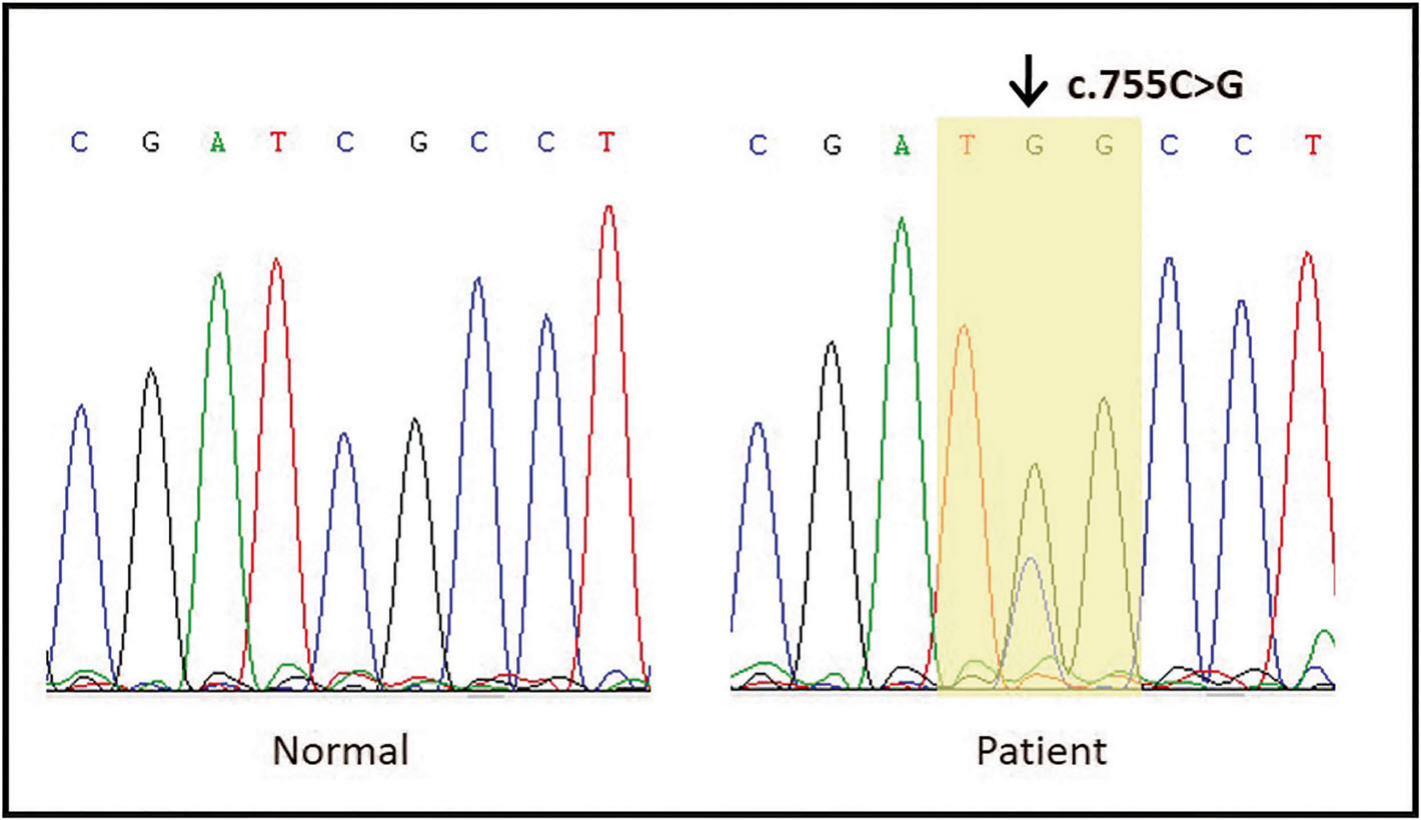
Partial sequence of exon 7 in *FGFR2* gene. *Arrow* indicates in the electropherogram the c.755G>C nucleotide change, compared with normal *FGFR2* gene DNA sequence. *Yellow shading* indicates consecutive codon change (p.Ser252Trp)

Mutation analysis was conducted for our patient. DNA was isolated from peripheral blood using the salt saturation method, as previously described by Miller *et al.* [15]. Molecular genetics analysis of *FGFR2*, targeting exon 7, was performed by direct sequencing at the Laboratory of Neurovascular Unit and Cognitive Impairments, University of Poitiers, France. The reference genomic DNA sequence used was NM_000141.4. Polymerase chain reaction (PCR) amplification of exon 7 from the *FGFR2* gene was performed using the primers *FGFR2*-F 5-CCGGCAGTCTCCTTTGAAGT-3′ and *FGFR2*-R 5′-GATCTGTTAATTCCTTAGAACACTCTCT-3′, resulting in a 525 bp fragment. Approximately 50 ng of DNA solution (2.5 μl) was added to 22.5 μl of PCR mixture. This PCR mixture contained 0.25 μl of 25 mM deoxyribonucleotide triphosphates (dNTPs), 3 μl of 25 mM MgCl_2_, 0.25 μl of each 20 μM primer, 2.5 μl of 10× PCR buffer, 0.125 μl of 5 U/μl Diamond® high fidelity *Taq* DNA polymerase (Eurogentec), and 16.13 μl of H_2_O. PCR was initiated with denaturation at 95 °C for 3 minutes, followed by 35 PCR cycles (at 95 °C for 30 seconds, 60 °C for 30 seconds, and 72 °C for 30 seconds) and 7 minutes final elongation at 72 °C. The amplified products were detected by electrophoresis on a 1.5% agarose gel with 0.5 mg/ml ethidium bromide and visualized under ultraviolet (UV) light. Furthermore, 5 μl of the PCR product was cleaned up with 2 μl ExoSAP reagent (ThermoFisher) according to the manufacturer’s instructions, to remove excess primers and unincorporated nucleotides enzymatically. Finally, 2 μl of the PCR product was used for the sequence reaction (BigDye Terminator Cycle Sequencing Kit Version 3.3; Applied Biosystems), which was run on an ABI PRISM® 310 Genetic Analyzer (Applied Biosystems), following the manufacturer’s directions. Sequencing was performed bidirectionally using the forward and reverse PCR primers. The sequence result was compared with the published reference sequence using Chromas software version 2.6.4. In this patient, we detected a missense mutation, changing a TCG codon (coding for a serine) into a TGG (coding for a tryptophan): p.Ser252Trp (c.755C>G) (Fig. 4).

## Discussion and conclusions

This is a case report describing an Indonesian patient with AS confirmed by molecular genetic analysis. The presence of the *FGFR2* mutation, p.Ser252Trp, was consistent with the diagnosis of AS. This mutation introduces additional affinity between FGFR2 and fibroblast growth factor 2 (FGF2). Consequently, gain-of-function interaction would lead to aberrant signaling, which would explain patient manifestations [16]. Due to unavailability of parental DNA, the mutation could not be determined as *de novo*. However, since the parents and both his siblings had no features of AS, this mutation apparently appeared *de novo*. Approximately 85% of cases of AS are caused by this missense mutation. However, this frequent mutation is not generalized for all cases of AS since at least seven mutations in the *FGFR2* gene have been found to cause AS [12, 13]. Subsequently, an advanced investigation of the whole gene is required with the aim of finding mutations that are possibly particular to the Indonesian population [17].

This case shows the common characteristics of patients with AS. There is acrocephaly, asymmetrical flat facies, and depressed nasal bridge with nasal deformity. The maxillary arch is V-shaped and sagittally narrow. Moreover, this patient displays severe dental crowding, delayed tooth eruption, and thick gingiva, which are common features of AS. However, tooth numbers are normal and not supernumerary. This patient also shows skeletal abnormalities (mild scoliosis and mild pectus excavatum), bilateral symmetrical syndactyl of both his feet and hands, and skin manifestations such as hyperhidrosis and hypopigmentation. Ocular proptosis, down-slanting of palpebral fissures, and hypertelorism are present, due to shortening of the bony orbit. It has been previously described that strabismus is more common in patients with the *FGFR2* p.Ser252Trp mutation and that preaxial polydactyly is also typical of this missense mutation [7, 18]. Our patient has strabismus and preaxial polydactyly, which is consistent with the clinical manifestations of the p.Ser252Trp mutation described in AS. However, there is no cleft palate which is statistically more prevalent in patients with p.Ser252Trp mutation [19].

Moloney *et al.* proposed a significant correlation between PAE and p.Ser252Trp mutation, which is in the context of a CpG dinucleotide [20]. Moreover, Glaser *et al*. described that contributing factors to the PAE may include selection and a higher number of mutant sperms [9]. In this case, our patient’s father was 37-years old, an increased mutation frequency due to older paternal age might be possible.

Varying severity of intellectual disability has been related with AS. It has been published that 52% of patients have an intelligence quotient (IQ) lower than 70, even if individuals with normal or borderline IQ have also been reported [21]. It has been assessed that this patient has borderline IQ that causes several problems, such as speech difficulties, attention deficit, and social problems.

A definite diagnosis of AS should be made by molecular DNA testing. However, AS can be clinically diagnosed and managed without molecular diagnostics, especially in developing countries with limited funding and lack of diagnostic services, in order to prevent late diagnosis and to enable early intervention. Treatment of a patient with AS should begin at birth within a comprehensive multidisciplinary care unit, including craniofacial, surgical, and developmental assessment. A craniectomy was performed on this patient when he was 3-years old. However, this procedure is often performed before 6 months of age to treat craniosynostosis and this may improve intelligence. Otherwise, syndactyly or webbing of fingers causes immobility of fingers following ossification of interphalangeal joints due to segmentation of embryonic phalanges. Therefore, this patient had corrective surgery twice at the age of 4 years on both hands to correct joint contractures. Corrective surgery for syndactyly should be done in the first year of life and completed by 3 to 4 years of age [22]. Moreover, counseling for the patient should involve referral to orthodontic and orthognathic surgery for a treatment plan. Genetic counseling is very important since recurrence risk in an autosomal dominant disorder for an affected individual to have an affected offspring is 50%.

In summary, we report the case of an Indonesian man with AS with a c.755C>G (p.Ser252Trp) mutation in *FGFR2* gene. Our patient showed similar dysmorphism to previously reported cases although a cleft palate, which is a typical feature of p.Ser252Trp mutation, was not present. In spite of the accessibility of molecular genetic testing in a few parts of the world, the acknowledgement of clinically well-defined syndromes will remain exceptionally imperative in developing countries with a lack of diagnostic facilities.

## Data Availability

All data generated or analyzed during this study are included in this published article.

## Abbreviations

AS: Apert syndrome
*FGFR2*: Fibroblast growth factor receptor 2
IQ: Intelligence quotient
OMIM: Online Mendelian Inheritance in Man
PAE: Paternal age effect
PCR: Polymerase chain reaction
